# Supplementary material for: Dynalign II: common secondary structure prediction for RNA homologs with domain insertions
Source: Nucleic Acids Res. 2014 Nov 21;42(22):13939–48. doi: 10.1093/nar/gku1172 (PMC4267632; doi:10.1093/nar/gku1172)
Supplement: SUPPLEMENTARY DATA [file supp_gku1172_nar-02021-z-2014-File012.zip › manual/Text/ShapeKnots.html]

RNAstructure Command Line Help: Fold


|  |  |  |
| --- | --- | --- |
|  | RNAstructure Command Line Help  ShapeKnots | - Contents - Index |
| ShapeKnots is used to predict a set of structures that can contain pseudoknots. It can take SHAPE mapping data as a restraint to increase structure prediction accuracy. USAGE: ShapeKnots <seq file> <ct file> [options] Required parameters: <seq file> \| The name of a sequence file containing input data. Note that lowercase nucleotides are forced single-stranded in structure prediction.; <ct file> \| The name of a CT file to which output will be written. Options which don't require added values: -h, -H, --help \| Display the usage details message.; -v, -V, --version \| Display version and copyright information for this interface. Options which require added values: -dsh, -DSH, --DSHAPE \| Specify a differential SHAPE data file to be used to generate restraints in addition to SHAPE restraints specified by --SHAPE. These restraints specifically use SHAPE pseudoenergy restraints where the offset is zero. Also, the function for calculating the pseudo free energy for nucleotide i is = (differential slope) \* (differential SHAPE for nucleotide i). These pseudoenergies are added to those generated with the --SHAPE option. Default is no differential SHAPE data file specified.; -dsm, -DSM, --DSHAPEslope \| Specify a slope used with differential SHAPE restraints. Default is 2.11 kcal/mol.; -dso, -DSO, --doubleOffset \| Specify a double-stranded offset file, which adds energy bonuses to particular double-stranded nucleotides. Default is to have no double-stranded offset specified.; -im, -IM, --IMaximum \| Specify a maximum number of internally generated structures for each call of the dynamic programming algorithm. Note that suboptimal structures are generated until either the maximum number of structures is reached or the maximum percent difference is reached (below).  This is not the maximum number of structures provided to the user, which is controlled by –m, -M, --maximum. Default is 100 structures.; -ip, -IP, --IPercent \| Specify a maximum percent difference in folding free energy change for internally generated suboptimal structures for each call of the dynamic programming algorithm. For example, 20 would indicate 20%. This is not the maximum percent difference in energy for structures provided to the user, which is controlled by –p, -P, --percent. Default is 20%.; -iw, -IW, --IWindow \| Specify a window size for the internally generated suboptimal structures for each call of the dynamic programming algorithm.  This is not the window for structures provided to the user, which is controlled by –w, -W, --window. Default is determined by the length of the sequence.; -m, -M, --maximum \| Specify a maximum number of structures. Note that suboptimal structures are generated until either the maximum number of structures are reached or the maximum percent difference is reached (below). Default is 20 structures.; -p, -P, --percent \| Specify a maximum percent difference in folding free energy change for generating suboptimal structures. For example, 20 would indicate 20%. Default is determined by the length of the sequence.; -p1, -P1, --Penalty1 \| Specify a pseudoknot penalty 1. Default is 0.35 kcal/mol.; -p2, -P2, --Penalty2 \| Specify a pseudoknot penalty 2. Default is 0.65 kcal/mol.; -ph, -PH, --PseudoknottedHelices \| Specify maximum number of helices to be processed. Default is 100 helices.; -sh, -SH, --SHAPE \| Specify a SHAPE data file to be used to generate restraints. These restraints specificially use SHAPE pseudoenergy restraints. Default is no SHAPE data file specified.; -si, -SI, --SHAPEintercept \| Specify an intercept used with SHAPE restraints. Default is -0.6 kcal/mol.; -sm, -SM, --SHAPEslope \| Specify a slope used with SHAPE restraints. Default is 1.8 kcal/mol.; -sso, -SSO, --singleOffset \| Specify a single-stranded offset file, which adds energy bonuses to particular single-stranded nucleotides. Default is to have no single-stranded offset specified.; -w, -W, --window \| Specify a window size. Default is determined by the length of the sequence. References: 1. Hajdin, C.E., Bellaousov, S., Higgins, W., Leonard, C.W., Mathews, D.H. and Weeks, K.M. “Accurate SHAPE-directed RNA secondary structure modeling, including pseudoknots.” *Proc. Natl. Acad. Sci. U.S.A.*, In Press. (2013). 2. Reuter, J.S. and Mathews, D.H. "RNAstructure: software for RNA secondary structure prediction and analysis." *BMC Bioinformatics*, 11:129. (2010). 3. Deigan, K.E., Li, T.W., Mathews, D.H. and Weeks, K.M. "Accurate SHAPE-directed RNA structure determination." *Proc. Natl. Acad. Sci. U.S.A.*, 106:97-102. (2009). | | |
| Visit The Mathews Lab RNAstructure Page for updates and latest information. | | |
